# An Introduction and Overview of RON Receptor Tyrosine Kinase Signaling

**DOI:** 10.3390/genes14020517

**Published:** 2023-02-17

**Authors:** Brian G. Hunt, Levi H. Fox, James C. Davis, Angelle Jones, Zhixin Lu, Susan E. Waltz

**Affiliations:** 1 Department of Cancer Biology, University of Cincinnati College of Medicine, Cincinnati, OH 45267-0521, USA; 2 Research Service, Cincinnati Veterans Affairs Hospital Medical Center, Cincinnati, OH 45220, USA

**Keywords:** RON receptor, MST1R, HGFL, macrophage stimulating protein, receptor tyrosine kinase

## Abstract

RON is a receptor tyrosine kinase (RTK) of the MET receptor family that is canonically involved in mediating growth and inflammatory signaling. RON is expressed at low levels in a variety of tissues, but its overexpression and activation have been associated with malignancies in multiple tissue types and worse patient outcomes. RON and its ligand HGFL demonstrate cross-talk with other growth receptors and, consequentially, positions RON at the intersection of numerous tumorigenic signaling programs. For this reason, RON is an attractive therapeutic target in cancer research. A better understanding of homeostatic and oncogenic RON activity serves to enhance clinical insights in treating RON-expressing cancers.

## 1. Genetic Overview of RON

The RON gene, *MST1R*, locus in humans is located on 3p21.31 [1]. This protein-coding gene contains 20 exons with multiple splice variants. Generally, RNA and protein expression of RON is low in most normal tissues for homeostatic balance. Proposed mechanisms of RON expression include transcriptional activation due to factors such as HIF-1a or NFkB, which are known to bind to the promoter of RON [2]. Nonetheless, the occurrence and outcomes of RON overexpression in various tissues are well-reported [3]. Conversely, suppression of the RON gene has been identified in nasopharyngeal carcinoma, where copy number changes and hypermethylation of CpG sites are correlated with decreased RON expression suggesting epigenetic mechanisms for the regulation of RON expression [4]. Together these patterns suggest a complex and dynamic role in the gene regulation of RON in a variety of tissue types resulting in distinct phenotypic outcomes described later in this Review.

Hepatocyte Growth Factor-Like (HGFL; gene name, *MST1*) is a ligand for RON. Interestingly, HGFL is genetically located in proximity to the RON locus on 3p21.31 [1]. This genetic proximity seems to be conserved in mice as the RON/*MST1R* and HGFL/*MST1* gene loci are both found on 9qF1 [5]. Moreover, analysis of The Cancer Genome Atlas (TCGA) Pan-Cancer datasets yielded a positive correlation between the expression of RON and HGFL in nearly all cancer types [3]. This relationship, taken in conjunction with the genetic proximity of *MST1R* and *MST1*, suggests the possibility of coregulation between these genes that, when disrupted, can manifest in physiologic consequences.

## 2. Structural Overview of RON

RON is in the MET family of receptor tyrosine kinases (RTKs) involved in cellular signaling in response to growth factors. Following maturation, RON is a single-pass, membrane-bound protein that serves to bind extracellular ligands and mediate intracellular responses. The translation product of RON-encoded mRNA begins as a glycosylated 185 kD polypeptide precursor and, like MET, requires proteolytic processing for signaling activity [6]. This precursor is cleaved, producing a heterodimer of the 35 kDa N-terminal extracellular α subunit and a 150 kDa transmembrane β subunit joined by a disulfide linkage [7]. The extracellular domain α subunit of RON is responsible for ligand binding with the β subunit of HGFL, which is contrasted with the extracellular domain of MET, which binds the α subunit of HGF [8]. While these pathways share degrees of homology, a further structural analysis may also be able to discern the distinct differences between the signaling mechanisms of RON and MET.

In canonical RON signaling, binding of HGFL to the extracellular portion of RON is sufficient to cause RON dimerization, leading to conformational changes conducive to autophosphorylation. Autophosphorylation is accomplished by the tyrosine kinase (TK) domain located on the intracellular region of the β subunit of RON [9]. The TK domain is a critical mediator of the downstream cascade and subsequent biological consequences. Autophosphorylation occurs on tyrosine residues Y1238 and Y1239 within the kinase domain of the β chain [10]. These activating phosphates then lead to kinase phosphorylation of tyrosine (Y) residues Y1353 and Y1360 on the C-terminus of RON’s intracellular tail. These phosphates serve as Src Homology 2 (SH2) or Phosphotyrosine-binding (PTB) docking sites for adaptor proteins that mediate the passage of signals to a variety of effectors for downstream signaling [11]. The SH2 domain of growth factor receptor bound 2 (GRB2) associates with these phosphorylated residues and is subsequently phosphorylated, allowing association with phosphatidylinositol 3-kinase (PI3K) which mediates AKT signaling [12]. Additionally, Son of Sevenless (SOS) can associate with GRB2 resulting in Ras activity and mitogen-activated protein kinase (MAPK) signaling driving proliferation [13,14].

Mutation of the C-terminal tyrosine residues has been shown to abrogate HGFL-induced signaling [12]. Additionally, in instances of signaling competent truncated RON, the intracellular domain is preserved for this activity [15]. Consistently, the tyrosine residues of the RON kinase domain and C-terminal tail have proven to be critical sites for propagating RON signaling through interactions with protein effectors.

## 3. RON Structural Variants

RON possesses several structural variants that are likewise relevant to human disease. Alternative splicing of RON yields functionally different RON isoforms with distinct activation and signaling mechanisms. While most known RON isoforms are not well-characterized, some truncated and short-form RON isoforms have been implicated in a variety of malignancies, including pancreatic, breast, and gastric cancers. In pancreatic adenocarcinoma, the prominent RON isoforms tend to lack an extracellular immunoglobulin-plexin-transcription (IPT) domain resulting from alternative splicing of exon 5 and exon 6 [16,17]. The partial 5 partial 6 deletion (P5P6) and RON 165 isoforms expressed in pancreatic cancer show constitutive activation, independent of ligand binding, leading to cellular transformation through AKT/MAPK signaling [16,17]. Similar deletions due to alternative splicing of exon 5 and exon 6 are found in colon and breast cancer tissue, resulting in a constitutively active RON isoform known as RON160 [18,19]. Alternative splicing in this exon 5/6 region is readily observed with transcripts, including both insertions and deletions in a variety of tissues [18]. In breast and gastric cancer, an alternative transcriptional start site in exon 10 produces an N-terminal truncation of RON lacking the extracellular domain but retaining the cytoplasmic and transmembrane domains [20,21]. SfRON was first described due to its ability to confer sensitivity to Friend virus-induced erythroleukemia [22]. Later studies implicated the physiological role of sfRON in regulating the mammalian immune response by attenuating IFN-y to mitigate acute liver injury [23]. Similar to the RON 160/P5P6 isoforms, this short-form RON (sfRON) is shown to be constitutively active but driving tumorigenic activity through different signaling mechanisms, including PI3K and B-catenin signaling [20,21]. This 55 kDa sfRON is accordingly associated with worse clinical outcomes, such as metastasis and therapeutic resistance [24,25]. It has been postulated that monoclonal antibodies targeting RON are unable to block sfRON due to the large structural differences between sfRON and wild-type RON [24]. While some small molecule RTK inhibitors have similarly shown poor efficacy in inhibiting sfRON [25], BMS777607-mediated RON-inhibition has demonstrated promising preclinical results in targeting sfRON [24,26]. While the sfRON and P5P6 isoforms are better understood, other RON isoforms have been identified but remain functionally unclear [19]. These isoforms and others are likely involved in RON’s tumorigenic role, and their structural differences from wild-type RON pose therapeutic challenges that require further insights.

## 4. Non-Canonical RON Signaling Mechanisms

While HGFL is the most extensively characterized ligand for RON, recent studies have suggested that RON can still have tyrosine kinase activity independent of HGFL binding [27]. Known mechanisms of HGFL-independent RON activation include heterodimerization with other RTKs (such as EGFR [28] and IGFR [29]), spontaneous dimerization due to RON overexpression [2], and receptor truncation [30]. Such conditions can produce effectively constitutively active RON signaling, which is further enhanced by HGFL ligand binding, driving tumorigenesis in many tissues [2]. This multi-faceted nature and degrees of RON activation and signaling demonstrate that further investigation is required in the pursuit of therapeutic intervention.

Heterodimerization of RON with differing binding partners allows for an expansion of signaling pathways and cellular functions. While RON and EGFR have demonstrated synergistic signaling enabled by heterodimerization [28,31], the novel transcriptional role of RON has also been uncovered [32]. In bladder cancer cells, ligand-independent RON in complex with EGFR migrated to the nucleus under serum starvation conditions. In the nucleus, the RON/EGFR complex bound and regulated the expression of genes governing cellular stress response, conferring a survival advantage [32]. Similarly, in prostate cancer cells, nuclear RON and transcription of c-FLIP promote cell survival [33]. These studies and others have noted a putative transcription factor consensus sequence for RON (5′-GCA(G)GGGGCACG-3′) under cellular stress conditions [32,33,34]. In this way, RON mirrors other RTKs, including MET, which demonstrates direct functioning in the nucleus [35,36]. Despite these findings, the nuclear role of RON is undefined and is seemingly context-dependent. Future studies are warranted to better characterize this direct gene regulation by RON and define clinical significance. 

## 5. RON Signaling in Macrophages and Other Bone Marrow-Derived Cells

RON is expressed in resident peritoneal macrophages and megakaryocytes but not in exudate macrophages or mononuclear phagocytes from bone marrow, peripheral blood, spleen, or alveoli [37,38]. As exudate macrophages become resident peritoneal macrophages, RON expression increases. In megakaryocytes, RON is associated with increased proliferation and IL-6 secretion, potentially linking to wound healing. Bone marrow mesenchymal stromal cells express RON and PDGFRA and have increased levels of MET, AXL, EGFR, and PDGFRB compared to hemopoietic stem/progenitor cells [39].

Activation of RON by HGFL in macrophages inhibits iNOS and IL-12 expression induced by LPS and IFN-y and LPS-induced apoptosis, as well as preventing NF-kB translocation to the nucleus [40,41,42,43]. RON activation in macrophages led to increased Arginase-1 expression, an important gene in the anti-inflammatory (M2) activation of macrophages, supporting that RON plays a role in macrophage polarization [44]. The inhibition of iNOS production and apoptosis in response to endotoxins is dependent on PI3K activity. RON activation promotes anti-inflammatory pathways, and loss of RON has been shown to result in enhanced LPS-endotoxin response and increased susceptibility to infection with Listeria monocytogenes [45]. In addition, RON activation in macrophages leads to reduced LPS-induced Cox2 expression and decreased HIV-1 viral transcription from decreased NF-kB activation [46,47]. Since both Listeria monocytogenes and HIV-1 infection can be impacted by RON, RON may play a role in susceptibility to subcellular infections.

Low-dose LPS treatment in mice resulted in less expression of RON in peritoneal macrophages, which appears to be NO-mediated RON suppression [48]. However, complicating the narrative, others have shown that LPS increased RON expression in primary macrophages [49]. RON TK^−/−^ mice lack the TK domain of RON and are overtly normal. However, RON TK^−/−^ mice exhibit increased susceptibility to endotoxin from enhanced IFN-y signaling, potentially from decreased DC maturation [50], as well as increased susceptibility to acetaminophen-induced hepatotoxicity [51]. LPS-induced Lipocalin 2 (Lcn2), a proinflammatory gene, is suppressed by HGFL-supplementation in RON TK^+/+^ control mice but not RON TK^−/−^ mice [52]. In addition, RON TK^−/−^ alveolar macrophages produce high levels of TNFa compared to that of RON TK^+/+^ control mice in response to LPS-mediated lung injury [53]. Interestingly, RON suppresses the TLR4-associated IFN signature only in FVB macrophages and not C57BL6 macrophages [54]. This shows the complex role genetics may have on differential RON activity and the need for multiple genetic models to accurately achieve the complexity of study that can be applied to a diverse group of humans.

## 6. RON Signaling in Reproduction and Development

In the developing embryo, RON is expressed starting in the nerves before being expressed in the heart, tongue, bones, and gut [55]. RON is detected in trophoblasts in the implantation stage of embryonic development [56]. RON^−/−^ mouse embryos, with a large targeted deletion of the RON gene locus, have been reported to fail to progress past the peri-implantation stage and are embryonic lethal by E6.5. Conversely, others have made a RON^−/−^ mouse line without any survival disadvantage [45]. These conflicting findings suggesting potential differences may be due to targeting strategies in a gene dense region or due to possible functions of non-kinase domain *RON* gene products [45]. HGFL^−/−^ mice reproduce and develop normally [57], functionally supporting ligand-independent functions. RON TK^−/−^ mice, which lack the RON TK domain, produce viable, fertile offspring [58].

RON also has potential roles in other aspects of reproduction that are not yet fully understood. Immature TK^−/−^ mice have decreased ovary size and decreased ovulation rates, as well as increased iNOS in the ovaries [59]. RON is expressed in spermatogonia, spermatocytes, and mature sperm [60], but the comparable litter sizes suggest it is dispensable for sperm development and function. In adult mice, RON is expressed in the uterus, placenta, liver, colon, epididymis, and testes and absent in the cervix, while HGFL is expressed in the placenta, cervix, liver, colon, epididymis, and testes [56]. This implies that RON may play a role in modulating NO and anti-inflammatory responses in various tissue types but is not required for cell type specification. In postnatal mammary gland development, HGFL^−/−^ mice have mammary gland defects such as smaller and fewer terminal end buds and delayed duct growth during puberty but do fully develop over time [61]. Levels of RON have been found to be increased in normal endometrial cells from women with deep endometriosis compared to healthy women as well as in invasive endometriosis cells compared to normal endometriosis cells, which implies that RON may play a role in the invasion of other tissues with uterine cells in endometriosis [62].

## 7. RON Signaling and Inflammation

RON and HGFL have been shown to be important in modulating inflammation in various tissue types. RON is expressed in response to DSS-induced colitis and decreases inflammation via the tyrosine kinase domain [63]. In addition, Qingchang Wenzhong Decoction, a Chinese herbal formulation, has been shown to improve inflammation in DSS-induced colitis in a mechanism requiring upregulation of RON and HGFL [64]. Interestingly, a coding variant of HGFL is associated with an increased risk of ulcerative colitis and Crohn’s disease, and in Zebrafish, an HGFL mutation results in spontaneous intestinal inflammation [65,66,67,68]. Similarly, LPS/GalN-induced inflammatory liver failure in RON TK^−/−^ mice showed decreased hepatocellular apoptosis. A follow-up study identified a novel signaling mechanism by which the RON receptor regulates liver failure progression requiring a JAK-STAT-mediated increase of the Suppressor of Cytokine Signaling (SOCS), which inhibits the production of inflammatory cytokines in Kupffer cells [69]. RON receptor tyrosine kinase also regulates the response to acute lung injury induced by nickel or intrapulmonary administration of LPS [70,71]. In RON TK^−/−^ mice, lungs showed clusters of macrophages, T cells, and neutrophils near the vascular endothelium and airways, as well as increased activation of NF-kB, TNFα expression, and NO production [72]. Noteworthily, RON was determined to be one of six genes mutated in patients with inverse psoriasis, an autoimmune disease in which areas of the skin are inflamed [73]. However, the direct outcomes of this mutation of RON have yet to be studied.

## 8. RON Signaling in Wound Healing

Wound healing involves the migration of epithelial cells and fibroblasts toward wounds. RON is expressed in fibroblasts and epithelial cells, and HGFL-treated fibroblasts and epithelial cells have increased migration [74,75,76]. The migratory effects of RON activation in keratinocytes, skin epithelial cells, are HGFL-PI3K-Integrin dependent [77,78]. There are increased levels of HGFL around wound sites, along with increased levels of RON in burn wounds [79]. There are higher levels of RON and HGFL in acute wounds than in chronic wounds [76]. In addition, wounds cause the pro-HGFL conversion to HGFL [79]. HGFL-treated mice have increased wound healing rates and collagen I and III production in fibroblasts, and HGFL has been shown to promote the adhesion of epithelial cells required to close wounds. [75,80].

In the kidney, HGFL expressed by tubular cells promotes growth, migration, and invasion of kidney mesangial cells [81]. In anti-Thy-1 nephritis, the elimination of HGFL led to a decreased influx of neutrophils and monocytes, lessened glomerular injury, and less mesangial cell overgrowth [82]. In addition, activated RON was observed in the glomerular lesions of patients with IgA nephropathy but not in other kidney diseases that do not involve mesangial cell growth [83]. These pieces of evidence imply that the RON/HGFL axis is important in mesangial cell regulation in response to injury. HGFL treatment of mice with gentamicin-induced renal tubule damage decreased inflammation and apoptosis, while HGFL treatment of renal proximal tubule cells decreased H_2_O_2_-associated apoptosis [84,85]. Further showing the importance of RON and HGFL in renal repair and survival, HGFL and RON upregulation has been observed in the regeneration phase after glycerol-induced tubular injury [86]. RON expression in mesenchymal stromal cells injected into a kidney transplant model of rats correlated with decreased rates of transplant rejection which implies that properties of RON in the milieu may be important in modulating host-transplant rejection [87].

## 9. RON Signaling in Ciliary Movement

RON is expressed in the apical cilia of the airway and the oviduct, and activation of RON by HGFL leads to increased ciliary beating [88,89]. This further illustrates the role that RON may play in reproduction. In addition, pro-HGFL is cleaved to HGFL at apical cilia via human airway trypsin-like protease [90]. Mutant RON is associated with an increased risk of Lady Windermere syndrome, and there are increased levels of HGFL in the sputum of patients with bronchiectasis [91,92]. These data suggest that RON activation may play an important role in the ciliary escalator to remove mucous from the lungs.

## 10. RON in Prostate Cancer

A transgenic mouse model with RON overexpression in the prostate epithelium [93] showed the development of prostate intraepithelial neoplasia (mPIN), along with the local invasion. Downstream signaling alterations are consistent with high RON activation, such as upregulated phosphorylation of ERK1/2 (p42/p44) and β-catenin. Using the Transgenic Adenocarcinoma of the Mouse Prostate (TRAMP) prostate tumor driver, TRAMP^+^ HGFL^−/−^ mice were found to have reduced genitourinary complex size and prostate size [94]. Histologically, the prostate tumors were similar in TRAMP^+^ mice. However, HGFL^−/−^ TRAMP^+^ mice had smaller genitourinary complex and prostate size as well as loss of STAT3 and Bcl-2, known downstream targets of RON signaling. These findings demonstrate the role of RON signaling within the prostate tumor microenvironment (TME) but do not clarify the role of individual cell types.

Further characterization of RON signaling in the myeloid compartment in supporting prostate tumor development was performed using a myeloid lineage-specific RON-deficient mouse model (RON TK^FL/FL^, LysM-Cre) [95]. Myeloid-specific RON loss showed reduced prostate cancer cell growth, reduced STAT3 activation, downregulated Arginase-1 expression (a marker of pro-tumorigenic macrophage polarization), and upregulation of iNOS (a marker of anti-tumorigenic macrophage polarization). Importantly, these effects on prostate tumor growth were rescued in mice that were subjected to bone marrow transplants from donor mice without myeloid deletion of RON. These effects were further shown to be dependent on T cell activity as T cell depletion groups resemble myeloid RON deleted groups. Examination of prostate epithelial RON expression recently published revealed a novel feed-forward loop wherein prostate epithelial RON signaling maintains RON expression in macrophages (which is of the myeloid lineage) [96]. Moreover, enhanced infiltration of TAMs and upregulated expression of M1 macrophage activation markers were observed. The mechanism by which prostate epithelial RON signaling drives macrophage-dependent RON expression and downstream effects on the tumor microenvironment and tumor growth has not been completely characterized.

One of the current first-line therapy for prostate cancer is androgen deprivation therapy (ADT). RON signaling has been shown to promote resistance to ADT through androgen-independent reactivation of the Androgen Receptor (AR) [97]. Interestingly, this mechanism is bolstered by RON-dependent CCL2 production and enhanced macrophage recruitment to the TME, which then provides GAS6 that serves to activate RON and Axl, another RTK, to promote ADT resistance [97]. Further considerations of this cellular crosstalk are detailed later in this Review.

## 11. RON in Breast Cancer

RON is overexpressed or constitutively active in >50% of human breast cancers. Using the Polyoma Middle T-antigen (PyMT) tumor driver crossed with RON TK^−/−^ mice (PyMT^+^, RON TK^−/−^), RON signaling was shown to be necessary for mammary tumor growth [98]. Additionally, RON overexpression in the mammary epithelium of mice (MMTV-RON) demonstrated the role of RON in mammary cell transformation, progression, and metastasis to the lung and liver in 100% of female mice [99]. These RON-driven mammary tumors showed a high degree of active β-catenin and upregulation of its targeted genes, Cyclin D1 and c-Myc. When β-catenin was conditionally deleted in mammary epithelial cells of MMTV-RON^+^ mice, decreased Cyclin D1 and abrogated metastasis was observed [100]. An interesting interaction between the Vitamin D receptor (VDR) signaling and RON signaling was observed wherein VDR signaling inhibits β-catenin activation in MMTV-RON^+^ mice [101]. These data support that β-catenin is a critical downstream effector for RON signaling in breast tumor growth and progression.

Tamoxifen is a drug commonly used in the treatment of hormone receptor-positive (HR^+^) breast cancer. However, its efficacy is limited by the development of drug resistance. Activation of the RON was shown to confer tamoxifen resistance in breast cancer cells, and HGFL-mediated RON activation can partially reverse tamoxifen-induced cytotoxicity in human and mouse breast cancer cell lines [102]. Additionally, when the target of tamoxifen, the estrogen receptor (ER), is deleted in RON overexpressing cells, the metastatic phenotype is enhanced while primary tumor growth is slowed [103]. The ER gene, *ESR*, is often mutated in ER^+^ metastatic breast cancer. A recent study using a Palbociclib resistance model showed that *ESR* mutant breast cancer had high RON signaling that was necessary for robust metastasis in a PI3K-dependent manner [104]. Together, these data suggest interactions between downstream signaling components of RON signaling and ER signaling that individually promote breast cancer. However, the exact positions of interaction remain uncharacterized.

HGFL^−/−^ mice were observed to show delayed but not a deficient extension of mammary ducts suggesting a role for HGFL-RON signaling in mammary stem cells [61]. Moreover, loss of STAT3 signaling was correlated with loss of RON activation in HGFL^−/−^ mice. Breast Cancer Stem Cells (BCSCs) play an important role in driving breast cancer initiation and progression due to their increased self-renewal, survival, and metastatic potential. HGFL-RON mediated non-canonical activation of β-catenin (phosphorylation of tyrosines 654 and 670) was found to drive enhanced BCSC self-renewal, enriched frequency of BCSC marker expressing cells, and enhanced tumor initiation under limiting dilution [105]. These data support that RON signaling and its downstream effector, β-catenin, drive BCSC phenotypes in aggressive breast cancer. Additional functions of RON signaling are provided by RON signaling in tumor-associated macrophages, which was shown to promote the production of IL-35, a member of the IL-12 family of cytokines, which was required for augmented BCSC self-renewal [106]. Thus, pro-BCSC functions of RON signaling have been demonstrated in at least two cell types of breast TME.

Loss of RON expression in macrophages showed reduced tumor growth kinetics and metastatic incidence in addition to altered BCSC self-renewal that also correlated with enhanced M1 macrophage marker expression, reduced M2 marker expression, and reduced T cell recruitment to the TME [106]. Moreover, these results were mirrored in mammary epithelial-specific loss of RON suggesting comparable functions in these two cell types [107]. In addition to RON overexpression reported in the PyMT model [98], HGFL overexpression was recently reported and functionally characterized through a cross of HGFL^−/−^ mice with PyMT^+^ mice. Once again, reduced tumor growth kinetics and metastatic incidence, as well as enhanced M1 macrophage marker expression, reduced M2 marker expression, and reduced T cell recruitment to the TME, suggesting a critical role of HGFL in the activation of RON signaling and its phenotypes likely for both tumor cell and macrophage RON signaling [3]. Further use of orthotopic transplant models of RON-overexpressing murine breast cancer cells with and without HGFL knockout into HGFL^+/+^ and HGFL^−/−^ hosts further bolstered that HGFL overexpression in mammary tumors is largely supportive of these phenotypes and plays significant roles in altering the tumor cell and macrophage secretome in a pro-tumor manner [3]. Further considerations regarding the cellular crosstalk within the TME are detailed later in this Review.

The PyMT model has additionally been used to characterize the direct effects of RON signaling on mammary tumor immunogenicity. CD8^+^ T cell function was significantly dampened under RON inhibition conditions allowing for complete conversion of micrometastasis to macrometastasis [108]. Moreover, interferon production, cytokine production, and immune signaling cascades were recently shown to be suppressed in RON-overexpressing breast cancer cells, and RON signaling allows for resistance to Natural Killer (NK) cell killing [107]. Interleukin 1 receptor-associated kinase 4 (IRAK4), a critical upstream mediator of pathways leading to type I interferon production, was found to directly associate with RON in this study, suggesting that RON has a direct suppressive function over pathways that promote cellular immunogenicity. Immune checkpoint inhibitors, a relatively new class of anticancer therapeutics that function by blocking mechanisms of T cell inhibition frequently found in cancer, were also shown to have enhanced efficacy when combined with RON inhibition [26]. In summary, RON signaling promotes breast cancer progression through immune suppression via mechanisms still undergoing characterization.

## 12. Emerging Ligand Crosstalk

In breast cancer, HGFL and RON co-overexpression was shown to promote sustained pro-tumor function in both tumor cells and tumor-associated macrophages [3]. These data demonstrate an autocrine (tumor cell to tumor cell) and paracrine (tumor cell to tumor-associated macrophage) mechanism by which HGFL and RON co-overexpression exerts their pro-tumor function. Further, activation by tumor cell-produced HGFL promotes changes to the tumor cell and tumor-associated macrophage secretome, each supporting tumor growth. However, the importance of specific factors altered remains under characterized [3]. ERK1/2 activation of the MAPK signaling pathway was reported to be required for tumor cell-dependent migration of tumor-associated macrophages [3]. In prostate cancer, RON overexpression was reported to promote CCL2 production, a chemoattractant for macrophages [97].

**Figure 1:**
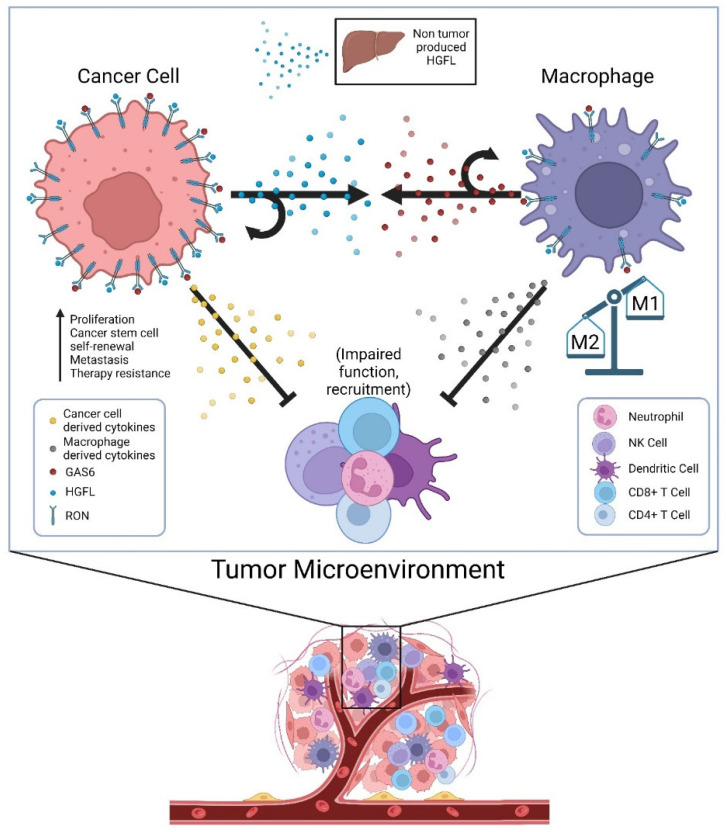
Cellular crosstalk of secreted factors in the tumor microenvironment (TME) that sustain high RON signaling in tumor cells and tumor-associated macrophages and alter the secretome to impair tumor immunogenicity. Physiologic sources of HGFL (primarily hepatocytes) provide a low level of HGFL for ligand activation, but tumor cells overexpress and produce high levels of HGFL to activate the abundant RON molecules on tumor cells in autocrine, and RON on the macrophages recruited to the TME. Tumor-associated macrophages secrete GAS6 providing its own means of RON activation on macrophages in autocrine and RON on tumor cells (and Axl where applicable). RON signaling in both tumor cells and tumor-associated macrophages alters the secretome of both cell types in manners that suppress innate and adaptive immune function, thus allowing for robust tumor growth.

Until recently, HGFL was thought to be the only known ligand of RON; now there is evidence showing that RON can also be activated by GAS6 [97]. GAS6 is a known ligand of the TAM receptor family, most specifically the Axl receptor [109]. GAS6/Axl signaling has been shown to activate many signaling pathways, including AKT, Jak/STAT, NF-kB, etc. and contribute to several oncogenic processes [109]. Though not much is known about GAS6 in the context of RON signaling, GAS6/Axl has been studied in several cancers, including the breast and prostate [109]. Given that RON signaling in prostate cancer cells promotes CCL2 production, which recruits tumor-associated macrophages, the secretion of GAS6 by tumor-associated macrophages to then promote RON activation adds an additional layer of cellular crosstalk to sustain RON signaling within the TME [97]. While GAS6-mediated activation of RON has not yet been reported in breast cancer, its effects on resistance to ADT in a RON-dependent manner in prostate cancer suggests at least some overlapping function of ligand-dependent activation irrespective of whether the ligand is HGFL or GAS6. Noteworthily, Axl expression is required for the most robust growth of RON overexpressing prostate cancer cells under androgen deprivation conditions; however, Axl activation does not appear to be sufficient alone [97]. A graphical summary of proposed cellular crosstalk that supports robust RON signaling within the TME and functional outcomes is described in Figure 1.

## 13. Open Questions in the Field of RON Signaling

Repeatedly, RON signaling has been shown to promote wound healing and the resolution of inflammation. From a translational perspective, acute activation of RON signaling may provide a means to reverse or dampen inflammatory conditions. RON activation through HGFL poses challenges due to the proteolytic processing required for HGFL maturation. However, the prospective activation of RON signaling via GAS6, which does not require extracellular maturation steps, presents an alternative means of RON activation [110,111]. GAS6-mediated RON activation was first described in prostate cancer and has not yet been tested in comparison with HGFL activation in inflammation and wound healing models. Further experimentation to demonstrate suppression of inflammation by GAS6-mediated RON signaling comparable to HGFL-mediated RON signaling is warranted. Moreover, GAS6 mediates activation of other receptor tyrosine kinases, including Axl, Tyro-3, and Mer, that are expressed in macrophages, epithelial cells, and more cell types, thus complicating the specificity of receptor activation [110,111]. Thus, the existing repertoire of means for RON activation has expanded but requires further preclinical testing to inform the translational potential.

Despite the sequence homology shared between RON and MET, and the overlap in oncogenic function, there remains a disparity in the academic and clinical focus of these RTKs. While MET has been extensively studied and has largely been targeted for pharmaceutical therapies, RON has not attracted nearly enough attention. Nonetheless, RON signaling, when significantly upregulated drives cancer initiation and progression; thus, inhibition of RON and its downstream mechanisms is a prospective means for anti-cancer therapy in a variety of tissue types. While currently, active clinical trials directly and specifically targeting RON are limited [112], the prospect of utilizing multi-kinase inhibitors has shown promise. One such multi-kinase inhibitor is BMS777607, a selective small molecule inhibitor that targets the MET superfamily of kinases, including Axl, RON, and c-MET [113]. Preclinical data examining RON inhibition show promising results in several cancer types wherein BMS777607-mediated RON inhibition abrogates tumor growth, metastatic progression, and treatment resistance and boosts immune checkpoint inhibitor efficacy [3,26,108,114] in preclinical models. While these results are promising, and BMS777607 appears to be a safe pharmacological compound [115], clinical testing of the efficacy of BMS777607 has not yet been performed, nor is it in the recruiting phase as a RON inhibitor or otherwise. Including BMS777607, other known MET-family kinase inhibitors have shown efficacy in targeting RON, such as PHA-665752 [116], Crizotinib [117], and Foretinib [118]. Due to the availability of MET-targeted pharmaceutical therapies, the degree of homology between RON and MET, and the overlap of downstream signaling, many MET-family kinase inhibitors show promise for targeting RON. Elucidation of downstream mechanisms through targeting with these existing drugs may provide alternative means to target pertinent downstream pathway activation driven by RON signaling.

Recent studies have identified significant upregulation of several metabolic pathways, including central carbon metabolism, cholesterol biosynthesis, and metabolism-related signaling pathways by RON signaling, and significant downregulation of interferon production, cytokine production, and immunity-related signaling pathways by RON signaling [26,107,119]. Thus, cellular metabolism and suppression of immune signaling molecules represent downstream targets of RON signaling that may represent novel means to target RON signaling outcomes in cancer. Critical open questions requiring investigation to provide necessary preclinical data for translational studies include establishing the requirement of each pathway in RON-driven cancer phenotypes (tumor initiation, metastatic progression, etc.), pathway mechanisms linking RON receptor activation to changes in expression (e.g., downstream regulatory pathways leading to upregulation in transcription/translation), and preclinical efficacy testing of existing compounds that target these pathways (e.g., statins to target cholesterol biosynthesis).

## 14. Conclusions

RON signaling has distinct functional outcomes, largely supporting the resolution of inflammation and promotion of wound healing under normal physiology. In pathology, loss of RON signaling results in prolonged inflammation and incomplete wound healing that can compromise critical organ function. In cancer, RON signaling is aberrant and is co-opted to promote robust tumor cell survival and growth while simultaneously shutting down inflammatory/immunogenic pathways to promote immune evasion. RON signaling is also sustained within the TME in multiple cell types and through multiple nodes of upregulated signaling, including its well-characterized ligand, HGFL, as well as GAS6. Emerging areas of research with prospective clinical translation include RON inhibition in conjunction with existing cytotoxic anti-cancer therapies to promote sensitization and immunogenicity and to target CSC populations that drive cancer metastasis and recurrence.

## Data Availability

Not applicable.
